# AI-DBS study: protocol for a longitudinal prospective observational cohort study of patients with Parkinson’s disease for the development of neuronal fingerprints using artificial intelligence

**DOI:** 10.1136/bmjopen-2024-091563

**Published:** 2025-05-16

**Authors:** Mariëlle J Stam, Martijn G J de Neeling, Bart J Keulen, Deborah Hubers, Rob M A de Bie, Rick Schuurman, Arthur W G Buijink, Bernadette C M van Wijk, Martijn Beudel

**Affiliations:** 1 Department of Neurology, Amsterdam University Medical Centres, Amsterdam, Noord-Holland, The Netherlands; 2 Department of Neurosurgery, Amsterdam University Medical Centres, Amsterdam, Noord-Holland, The Netherlands; 3 Department of Human Movement Sciences, Faculty of Behavioural and Movement Sciences, Vrije Universiteit Amsterdam, Amsterdam, Noord-Holland, The Netherlands

**Keywords:** Parkinson-s disease, Neurophysiology, Observational Study

## Abstract

**Abstract:**

**Introduction:**

Deep brain stimulation (DBS) is a proven effective treatment for Parkinson’s disease (PD). However, titrating DBS stimulation parameters is a labourious process and requires frequent hospital visits. Additionally, its current application uses continuous high-frequency stimulation at a constant intensity, which may reduce efficacy and cause side effects. The objective of the AI-DBS study is to identify patient-specific patterns of neuronal activity that are associated with the severity of motor symptoms of PD. This information is essential for the development of advanced responsive stimulation algorithms, which may improve the efficacy of DBS.

**Methods and analysis:**

This longitudinal prospective observational cohort study will enrol 100 patients with PD who are bilaterally implanted with a sensing-enabled DBS system (Percept PC, Medtronic) in the subthalamic nucleus as part of standard clinical care. Local neuronal activity, specifically local field potential (LFP) signals, will be recorded during the first 6 months after DBS implantation. Correlations will be tested between spectral features of LFP data and symptom severity, which will be assessed using (1) inertial sensor data from a wearable smartwatch, (2) clinical rating scales and (3) patient diaries and analysed using conventional descriptive statistics and artificial intelligence algorithms. The primary objective is to identify patient-specific profiles of neuronal activity that are associated with the presence and severity of motor symptoms, forming a ‘neuronal fingerprint’.

**Ethics and dissemination:**

Ethical approval was granted by the local ethics committee of the Amsterdam UMC (registration number 2022.0368). Study findings will be disseminated through scientific journals and presented at national and international conferences.

**STRENGTHS AND LIMITATIONS OF THIS STUDY**

- A comprehensive observational, prospective, longitudinal study collecting data from 100 patients with Parkinson’s disease treated with sensing-enabled deep brain stimulation.
- A multimodal dataset, including kinematic, clinical, neuronal and diary data, will be gathered both in-clinic and in the patients’ home environment.
- Group-level correlations and patient-specific modelling will lead to the identification of neuronal profiles reflecting individual symptoms.
- Limitations include the potential lack of contextual understanding when collecting data from patients at home, potentially hindering data interpretation.

## Introduction

### Background and rationale

 Parkinson’s disease (PD) is a neurodegenerative disorder that leads to debilitating motor and non-motor symptoms. The characteristic motor symptoms of PD (bradykinesia, resting tremor, rigidity and postural instability) are due to degeneration of midbrain dopaminergic cells.^1^ In approximately the first 5–10 years of the disease, treatment with dopaminergic medication, such as levodopa, can substantially improve motor symptoms without complications.^2 3^ Unfortunately, the majority of patients will experience so-called motor response fluctuations with the advancement of the disease. In this phase, patients exhibit rapid, sometimes unpredictable swings between mobility (called the ON-drug phase), usually with dyskinesias, and immobility (called the OFF-drug phase). Many of these patients respond unsatisfactorily to adjustments of the regular pharmacological treatment.^4^

To manage medication-induced response fluctuations, deep brain stimulation (DBS) provides an alternative, advanced treatment option. The leading hypothesis about the mechanism of action of DBS is its modulation of pathological neuronal activity by delivering electrical stimulation through electrodes inserted in specific deep brain structures. DBS of the subthalamic nucleus (STN) has been proven to be an efficacious-advanced treatment for patients with PD with motor fluctuations.^5^,^7^ There is class I evidence that STN-DBS reduces the duration and severity of symptoms during the OFF-drug phase, reduces the duration and severity of dyskinesias and improves quality of life in patients with PD.^8^,^10^

At present, not every patient with PD with severe response fluctuations benefits from DBS treatment because of suboptimal motor improvement or the occurrence of side effects induced by the stimulation. This might be partly due to the current practice of applying stimulation continuously: high-frequency (80–185 Hz) stimulation is provided day and night and is only interrupted or adjusted by either the patient or by the physician during often labourious hospital visits to programme the DBS device based on subjective assessments.^11^ During this continuous form of DBS (cDBS), the stimulation does not adapt to the severity of symptoms that may fluctuate due to, for example, the intake of oral medication or other physiological changes that may occur. In theory, DBS might be more efficacious if it only emits current when necessary and if the current can be adjusted to the needs of the patient at a given moment or disease stage. Several factors support this assumption. First, DBS improves bradykinesia when present (during OFF-drug phase), yet it reduces motor velocity in the absence of bradykinesia (during ON-drug phase).^12^ In other words, DBS might be counter-productive during the ON-drug phase (ie, when symptoms are absent because dopaminergic medication is effective). The assumption is further supported by a line of successive experiments showing that selectively administering DBS current leads to less stimulation-induced side effects,^13^,^15^ such as dysarthria^15 16^ and dyskinesia,^17^ and lower battery consumption.^18^

The automatic adjustment of stimulation is referred to as ‘closed-loop’ or ‘adaptive’ DBS (aDBS) and uses physiomarkers that reflect the presence or severity of a symptom.^19^ In PD, neural oscillations in the beta frequency band (13–30 Hz) of local field potential (LFP) signals, measured with DBS electrodes, currently form the best candidate neurophysiological physiomarker for patients undergoing STN-DBS. Beta-band STN-LFP spectral power has frequently been shown to correlate with the severity of contralateral bradykinesia and/or rigidity.^20^,^23^ Furthermore, beta power decreases after dopaminergic medication is administered^14 24^ or after DBS is switched on.^25^ Other examples of physiomarkers are pallidal low-frequency (4–12 Hz) LFP oscillations in dystonia patients^26^ and accelerometer amplitude in tremor-dominant patients.^27^ It is important to realise that appropriate physiomarkers are likely to vary from patient to patient. In PD, for example, the specific frequency that correlates most strongly with symptoms such as bradykinesia or rigidity can vary within the beta range, with each patient exhibiting an individual so-called ‘beta peak’.^28^ Moreover, aDBS based on beta oscillations does not work as efficiently for tremor as it does for bradykinesia,^15^ and dyskinesias are more associated with alterations in the gamma band (>35 Hz).^29^ This highlights the need for a personalised algorithm that adjusts stimulation based on a patient-specific and symptom-specific profile of pathological neuronal activity, that is, a ‘neuronal fingerprint’.

Despite the many (theoretical) advantages of aDBS over the currently applied cDBS, almost all studies identifying and validating STN-LFP physiomarkers to date have been conducted in small groups of patients, performing only short recordings in controlled in-clinic experimental settings with externalised DBS electrodes, under controlled medication state, usually while patients were at rest and stimulation was turned off,^2530^,^32^ which makes it difficult to extrapolate the findings to the applicability in daily life. In 2020, Medtronic (Minneapolis, Minnesota, USA) released a fully implantable Conformité Européene (CE)-marked DBS system that is able to concurrently stimulate and to record LFP signals from the electrode contacts.^33^ This system, the Medtronic Percept PC neurostimulator, can facilitate aDBS care in out-of-hospital settings, in large cohorts of patients with PD and is currently being implanted in patients worldwide, including in the Amsterdam UMC. The first research studies were aimed at establishing the technical feasibility to chronically sense neuronal activity with a fully implanted DBS system.^34^,^36^ Initial findings from our research group have further confirmed this.^37 38^ However, in order to proceed to the clinical implementation of aDBS in PD, patient-specific and symptom-specific physiomarkers should be well established and their application outside controlled hospital settings should be studied.^39^

To facilitate the translation of the technical advancements to clinical implementation of aDBS, the artificial intelligence (AI)-DBS study will collect a high-resolution multimodal dataset of, to our knowledge, the largest cohort of patients with PD with sensing-enabled DBS to date, including recordings obtained in-clinic as well as during the patient’s daily living for over 180 days. The latter is an important aspect in order to overcome the challenge of developing an aDBS control system able to react to patients’ everyday actions, behaviours and other relevant states, such as ON–OFF medication and ON–OFF stimulation.^40 41^ A cohort of patients this large has the potential to unveil novel physiomarkers such as activity in other frequency bands alongside the well-established beta band or features in the time domain.^41^ We anticipate machine learning and deep learning techniques to be particularly useful for automatic discrimination between physiological and pathological neural activity.^40^ The methods also hold promise for advancing personalised treatment strategies, acknowledging the variability in disease symptoms across patients and the possibility of certain physiomarkers not being applicable in all patients. The use of machine learning and deep learning in the AI-DBS study allows for individualising the selection of (or the combination of) most effective or informative features concerning symptomatology or clinical status, hence following the concept of ‘personalised medicine’. The multifaceted data analysis approach employed in the AI-DBS study will enable the conduct of a future clinical trial comparing the neuronal fingerprint-based programming/DBS approach with conventional DBS, to further improve the clinical effect and efficiency of DBS for PD.

### Objectives

The primary objective of the AI-DBS study is to identify patient-specific profiles of pathological neuronal activity that are associated with the presence and severity of specific symptoms, forming a ‘neuronal fingerprint’. These fingerprints will be generated by correlating the patient’s neurophysiological signals with the severity of their motor symptoms and by using AI in an exploratory fashion. Secondary objectives include the investigation of DBS-induced changes in LFPs in relation to alterations in kinematic data and diary-reported symptoms, medication and stimulation parameters, as well as clinical outcomes such as severity of motor symptoms, quality of life and apathy. Finally, the study will explore whether LFPs can predict PD motor symptom severity and DBS side effects, as well as which DBS contact points are best selected for clinical practice. In doing so, the AI-DBS study will specifically fill gaps around personalising the approach towards aDBS therapy programming and targeting the benefits to individual patient needs.

## Methods and analyses

### Study design

The study is a prospective and observational study carried out in a tertiary referral centre for movement disorders in the Netherlands. Patients with PD eligible for DBS of the STN are invited to participate.

### Study population

100 patients with PD who will be implanted with the Medtronic Percept PC neurostimulator for STN-DBS in the *care as usual* setting will be included. Eligibility for DBS treatment is determined based on the occurrence of motor fluctuations and levodopa responsiveness, regardless of patients’ PD subtype. Only for diagnosing purposes, patients’ genetic factors are considered, but not for the decision on being selected for DBS.^42^ The DBS device will be programmed in a *care as usual* setting, which means that the participants will have to visit the hospital frequently for the gradual optimisation of stimulation parameters and medication schedule. To be eligible for inclusion, a participant must be 18 years or older, must be diagnosed with PD by their treating neurologist and eligible for DBS and must receive Medtronic Percept PC DBS of the STN between February 2022 and February 2025. A potential participant who is unable to provide informed consent will be excluded from participation in this study.

#### Recruitment

Eligible patients will be provided with a participant information sheet and given the opportunity to discuss the study with one of the investigators and to ask questions. In case the patient decides to participate, the patient as well as the investigator will sign the informed consent document. Participants have the right to withdraw consent at any time without citing reasons and without any consequences. Participants can withdraw consent for all study involvement, or from involvement in certain elements of the study.

### Data collection

The following types of data will be collected and analysed for all included participants

- Age, sex, year of symptom onset, neurological examination and neuroimaging.
- Smartwatch inertial sensor data (three-axis accelerometer and gyroscope).
- Kinematic tremor and dyskinesia probability scores.^43^
- PD severity, measured by the Movement Disorder Society—Unified Parkinson’s Disease Rating Scale (MDS-UPDRS)^44^ (MDS-UPDRS part III will be filmed).
- Health-related quality of life, measured by the Parkinson Disease Questionnaire—39 (PDQ-39) summary index.^45^
- Apathy, measured by the Starkstein Apathy Scale (SAS).^46^
- Depressive symptoms, measured by the Beck Depression Inventory (BDI).^47^
- Longitudinal passive at-home LFP recordings of a frequency of interest (BrainSense Timeline^48^).
- High-resolution LFP recordings during clinical visits (BrainSense Survey, Setup and Streaming^48^).
- Digital patient diary consisting of the time points of experienced bradykinesia, rigidity, tremor and dyskinesia, and their corresponding LFP data in the form of full-spectrum power spectral densities (PSDs) (BrainSense Events^48^).
- Clinical documentation of (changes in) medication and/or stimulation parameters.

#### Study procedures

**Figure 1:**
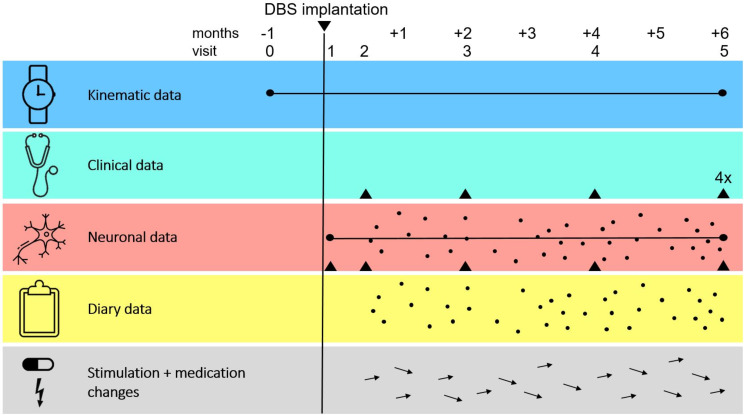
Schematic representation of the different forms of data collection during the study. The horizontal axis represents the approximate 7-month timeline during which a patient is enrolled in the study. The six study visits to the hospital are indicated at the top of the figure together with the moment of deep brain stimulation (DBS) implantation (black vertical line). Participants will wear a smartwatch with built-in inertial sensors throughout the entire study period from which estimates of tremor and dyskinesia severity are determined (black horizontal line in the blue row, ‘kinematic data’). Participants’ motor symptoms severity will be determined while the DBS is on and participants are on their regular medication during visits 2–4, and in four different conditions (DBS off/on and off/on medication) during visit 5 (black triangles in cyan row, ‘clinical data’). Local field potential recordings (red row, ‘neuronal data’) will be conducted from DBS implantation onwards, not only during visits 1–5 (black triangles) but also passively at home (black horizontal line) and at given moments throughout the day at home (black dots, moments are randomly indicated in the figure). Participants will indicate how they are feeling at these same specific moments of the day (black dots in the yellow row, ‘diary data’). Adjustments in stimulation parameters and/or medication schedule as part of care as usual DBS procedure will be registered throughout the study (moments are randomly indicated with the black arrows in the grey row, ‘stimulation+medication changes’).

A schematic overview of all data that will be collected is shown in figure 1. Written informed consent will be obtained 1 month before DBS surgery (visit 0), after which baseline characteristics will be collected from patient files. These include age, sex, symptom onset, neurological examination and neuroimaging. After visit 0, participants will undergo five testing visits. The first visit will take place 1 day after DBS surgery (visit 1). Visit 2 will occur when the DBS system is activated, typically 2 weeks after implantation (to avoid major stun effects^49^). Visits 3 and 4 (2 and 4 months post surgery, respectively) will often coincide with regular clinical visits, but may be scheduled as separate study visits if the time interval between standard care visits appears insufficient. The testing period will end at the moment of the regular clinical follow-up visit at approximately 6 months post surgery (visit 5).

##### Kinematic data

From the day of obtaining written informed consent (visit 0) until the participants’ regular clinical follow-up visit (visit 5) usually 6 months after surgery (7 months in total), kinematic data will be collected from a smartwatch at the wrist of the most affected body side of the participant (Apple Watch SE 2020 or 2022, Apple). The smartwatch has built-in inertial sensors (three-axis accelerometer and gyroscope with 100 Hz sampling rate) from which it is possible to estimate the severity of tremor and dyskinesia and potentially other movement parameters.^43 50 51^ These kinematic data will be collected through the Apple HealthKit repository and further processed with the StrivePD mobile app (Rune Labs) installed on a smartphone (iPhone 8 or equivalent, running at least iOS 16, Apple) that is paired with the participants’ smartwatch.

##### Clinical data

Participants will undergo clinical testing during visits 2–5. During visits 2–4, the severity of the participant’s motor symptoms will be determined using the MDS-UPDRS part III (motor) scores, while DBS is switched on and participants are on their regular medication. Apart from the items for rigidity, the MDS-UPDRS part III assessment will be filmed and rated afterwards. During visit 5, the effect of DBS will be evaluated systematically using MDS-UPDRS part III scores in four different conditions: off medication and DBS switched off (OFFmed/OFFstim), off medication and DBS switched on (OFFmed/ONstim), ONmed/OFFstim and ONmed/ONstim. In addition, MDS-UPDRS part I (non-motor symptoms), MDS-UPDRS part II (activities of daily living), MDS-UPDRS part IV (motor complications), the PDQ-39, SAS and BDI scores will be obtained. These questionnaires, as well as the MDS-UPDRS part III scores in two conditions (OFFmed and ONmed), are also available at the preoperative stage obtained in a *care as usual* way prior to the start of the study.

##### Neuronal and diary data

Neuronal and diary data will be collected using BrainSense Technology of the Percept neurostimulator.^33 52 53^ The day after DBS surgery (visit 1), the BrainSense Survey feature (Survey) will be used to obtain full-spectrum PSDs from each sensing-enabled contact pair estimated over LFP recordings of approximately 20 s (at 250 Hz sampling frequency) (ONmed/OFFstim). When using the Survey feature, stimulation is always switched off. Subsequently, the BrainSense Setup feature (Setup) will be used to estimate full-spectrum PSDs of each stimulation-compatible contact pair (estimated over LFP recordings of approximately 20 s at 250 Hz sampling frequency) (ONmed/OFFstim). Other than sensing without stimulation (sensing-enabled), concurrent sensing and stimulation (stimulation-compatible) is only possible when using a symmetric dipole (sandwiching) around the stimulation electrode contact point with monopolar stimulation referenced to the implanted neurostimulator.^54^ Therefore, only three combinations of contact pairs are stimulation-compatible. The PSDs obtained from both Survey and Setup recordings will be used to determine the frequency of interest setting for activation of the longitudinal passive LFP recordings, that is, BrainSense Timeline (Timeline). For each hemisphere, Timeline will be activated at the stimulation-compatible contact pair for which the PSD shows the most prominent peak in the beta-band frequency range (13–35 Hz). After participants leave the clinic, the Timeline feature will record the LFP signal (sampled at 250 Hz), convert it into the frequency domain using an on-chip ‘Fast Fourier Transform’ method (1 s Hanning window and 50% overlap) and store the average power within an approximately 5 Hz wide frequency band surrounding the frequency of interest over a 10 min interval on the Percept neurostimulator. After setting up Timeline, the BrainSense Streaming feature (Streaming) will be used to record the LFP signal of the previously chosen stimulation-compatible contact pair (at 250 Hz sampling frequency) at rest for 2 min, the first minute with the stimulation OFF, the second minute with the stimulation ON at 0 mA (ONmed/OFFstim). Leaving the stimulation ON at 0 mA, rather than switching it OFF, secures accurate comparison when the stimulation amplitude is increased in the following months. Recording the first minute with stimulation OFF allows us to study the difference with the DBS system ON at 0 mA, helping to identify potential artefacts and spectral content differences between the two recording modes relevant for the comparison with other study results.^33^

During visit 2, standard care procedures will be performed to determine the electrode contact used for stimulation. Prior to increasing the stimulation amplitude and administering dopaminergic medication, PSDs of all sensing-enabled contact pairs will be obtained again using the Survey feature (OFFmed/OFFstim). Subsequently, based on the electrode contact selected for stimulation, the Setup feature will be reused to activate the Timeline and Streaming functionalities for LFP recording of the surrounding contact pair (OFFmed/OFFstim). This also means that the stimulation-compatible contact pair for LFP recording through Timeline selected at visit 2 might differ from the contact pair used between visits 1 and 2. By means of the Streaming feature, the LFP signal will be recorded during 1 min rest with the DBS system still ON at 0 mA (OFFmed/OFFstim). After medication intake, the estimation of PSDs of all sensing-enabled contact pairs through the Survey feature will be repeated (ONmed/OFFstim). Standard DBS care procedures will then be followed in order to increase the stimulation. At the stimulation amplitude determined through *care as usual* procedures and while participants are on dopaminergic medication, the LFP signal will be recorded using Streaming during 1 min rest, followed by MDS-UPDRS part III assessment (approximately 10 min in total ONmed/ONstim).

During the following two testing visits (visits 3 and 4), the Streaming feature will be used to perform the same 10 min LFP recording (ONmed/ONstim) consisting of 1 min rest followed by the MDS-UPDRS part III. Visit 5 will coincide with the regular clinical follow-up visit. During this visit, the Survey feature will be used twice: before (OFFmed/OFFstim) and after (ONmed/OFFstim) medication intake. In addition, the 10 min LFP recording through Streaming will be performed four times while the effect of DBS is evaluated using the MDS-UPDRS part III in four conditions, as described in ‘clinical data’ above. A structured summary of the various BrainSense features used to collect the LFP data during the study visits is provided in table 1.

**Table 1:** Summary of BrainSense features used to collect local field potential data during study visits

	Visit 1	Visit 2	Visit 3	Visit 4	Visit 5
Survey: 220 s rest*	M^+^S^−^	M^−^S^−^M^+^S^−^			M^−^S^−^M^+^S^−^
Setup: 90 s rest†	M^+^S^−^	M^−^S^−^			
Streaming: 60 s rest‡	M^+^S^−^§	M−S^−^M^+^S^+^	M^+^S^+^	M^+^S^+^	M^−^S^+^M^−^S^−^M^+^S^−^M^+^S^+^
Streaming: UPDRS part III‡		M^+^S^+^	M^+^S^+^	M^+^S^+^	M^−^S^+^M^−^S^−^M^+^S^−^M^+^S^+^

* All sensing-enabled contact pairs.

† All stimulation-compatible contact pairs.

‡ Selected stimulation-compatible contact pair.

§ Both OFFstim and ONstim at 0 mA.

M, medication intake no (−) or yes (+); S, stimulation OFF (−) or ON (+).

**Figure 2:**
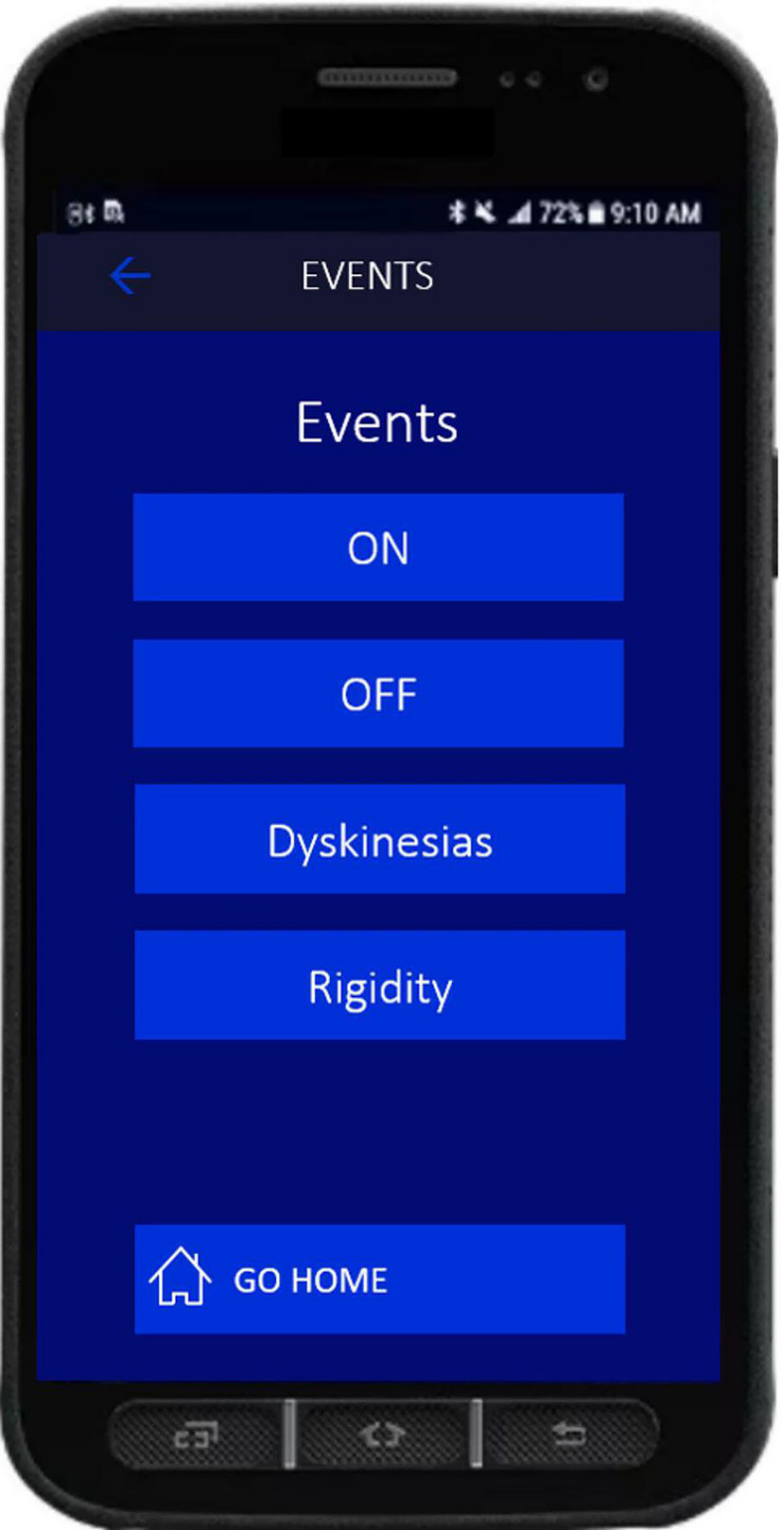
Patient programmer of the Percept PC showing the diary events that can be entered by the participant. The content of the events can be adjusted to the actual symptoms of the patient.

Other than the passive at-home LFP recordings between visits 1 and 5 using BrainSense Timeline, participants will be asked to use the BrainSense Events feature (Events) to fill in a diary five times a day from visit 2 onwards. In the context of standard DBS care, patients receive a smartphone of the Percept PC DBS system, called the ‘patient programmer’, allowing them to adjust their DBS parameters within the limits set by their healthcare provider. On receiving a reminder from their smartwatch, participants will be asked to use the patient programmer to indicate whether at that specific moment of the day they are feeling ‘OFF’, ‘ON’, or are suffering from either of two patient-specific symptoms (figure 2). As participants enter an Event, a full-spectrum PSD estimated over an LFP recording of 30 s (at 250 Hz sampling frequency) will be obtained from the stimulation-compatible contact pair used for LFP recording.

The contact pair used for LFP recording through the Streaming, Timeline and Events features will depend on the electrode contact selected for stimulation through standard DBS care procedure, which therefore may vary over the course of the study period. In fact, as concurrent sensing requires symmetric dipole (sandwiching) around the stimulation electrode contact point, selecting either the ventral or dorsal electrode contact for stimulation will preclude the activation of the Streaming, Timeline and Events features for that specific hemisphere.

##### Stimulation and medication changes

Throughout the course of the study, participants will undergo their DBS care in a *care as usual* way which implies regular contact with DBS nurse specialists, who, if necessary, make adjustments to medication and stimulation parameters using either the patient programmer remotely at home or during in-clinic visits. The clinicians programming the DBS will be blinded for the study findings. Their observations leading to changes in stimulation parameters will not be influenced by (interim) results of the study. Changes in medication will be stored in patient files. Changes in stimulation parameters will be stored in the Percept neurostimulator memory. Both will be made available for the AI-DBS study.

### Patient burden and safety

The administration of the different clinical scales in this study is part of the standard procedures in the Amsterdam UMC, and the study visits mostly coincide with regular clinical visits. Participation adds only slight discomfort of wearing a smartwatch, making small diary notes and undergoing a maximum of two extra testing visits. Therefore, the envisioned disadvantage of participation is limited. However, if patients experience excessive burden from participation, they also have the option to withdraw consent from certain elements of the study. The proposed protocol bears virtually no risks since readout of the neural recordings is safe and non-invasive. Although patients will not directly benefit from their participation, it is conceivable that in the medium to long term, the AI-DBS study might lead to improvements in aDBS treatment that will be to their benefit.

Adverse events (AEs) reported spontaneously by participants or observed by the study staff will be systematically recorded. Serious AEs (SAEs) associated with study-related procedures will be reported by the investigator without undue delay after obtaining knowledge of the events. SAEs resulting in death or considered life-threatening will be reported via the ‘ToetsingOnline’ web portal to the accredited medical research ethics committee that approved the protocol. The initial report must be submitted within 7 days of awareness, followed by an additional period of up to 8 days for a detailed preliminary report. All other SAEs will be reported within 15 days of first knowledge by the staff. AEs will be monitored until they resolve or stabilise. Follow-up actions may involve additional tests, medical procedures or referral to a general practitioner or specialist, according to the nature of the event. Reporting of SAEs is required until the end of the study within the Netherlands.

### Data analysis

#### Sample size

Recent examples of similar AI-based DBS studies include Habets *et al*^55^ (n=89), Peralta *et al*^56^ (n=84) and Yim *et al*^57 57^ (n=81). The sample size of the current study (n=100) is feasible because 3 years are envisaged for inclusion and approximately 100 patients with PD receive DBS in the Amsterdam UMC each year.

#### Analysis plan

The Streaming data obtained during the in-clinic visits will be preprocessed as described previously.^58^ The statistical analysis will be performed using R statistical software V.4.0.5 (R Core Team, Vienna, Austria), MATLAB versions R2022b/R2023b and Python V.3. In all analyses, a two-sided p value<0.05 will be considered statistically significant. Depending on the sample size of the data, either the Shapiro-Wilk test (sample sizes up to 50 samples) or the Kolmogorov-Smirnov test (sample size of 50 or more samples) will be used to test for normal distribution of the data. Descriptive statistics will be used to summarise baseline participant demographics. The main data analysis in this study is divided into two approaches, each addressing specific research objectives.

##### Temporal profile of LFP spectral power and relation to motor symptom severity

The first approach consists of assessing (changes in) the different data modalities (as described in ‘data collection’) and their correlations with (changes in) clinical outcome on a group level. Where appropriate, Z-transformation will be applied to both neuronal Timeline data and kinematic scores, to enable meaningful group-level correlations. Relative full-spectrum PSDs will be obtained by normalising the power at each frequency of the absolute full-spectrum PSD to the total summed power of that specific spectrum. Additionally, the contributions of individual participants will be balanced by down sampling, taking into account data availability (eg, the number of recorded Events and adherence to wearing the smartwatch) to avoid bias in group-level comparisons. A Pearson correlation will be performed for normally distributed data, and a Spearman correlation will be used for data that is not normally distributed. Changes in the LFP Streaming data between pre-DBS and post-DBS activation will be evaluated using Analyses of Variance (ANOVAs) (for normally distributed data) or a Kruskal-Wallis H test with Wilcoxon signed-rank for post hoc analysis of significant results (for non-normally distributed data) of spectral power in different frequency bands after calculation of the PSD per hemisphere. Correlations will be tested between the Timeline data obtained at home and the simultaneously obtained kinematic scores, as well as the diary data in the weeks and days prior to the clinical visits. Changes in the temporal structure of the corresponding Timeline data averaged per week, day and hour will be assessed using change point analysis. Furthermore, it will be explored how changes in medication and stimulation are reflected in the Timeline data, kinematic data and diary data. For this, time intervals (with incremental steps of 1 day, up to 2 weeks) before and after adjustments will be compared. Additionally, correlation analyses will be conducted to investigate associations between changes in LFP measures and changes in (contralateral) MDS-UPDRS part III (hemibody) scores and between LFP measures and the scores on the PDQ-39, SAS, BDI and MDS-UPDRS part I, II and IV obtained during visit 5.

##### Prediction of clinical DBS parameters and outcome, and creation of individual neuronal fingerprints

For the second approach, an AI-based analysis will be implemented for the identification of predictive LFP features. Based on the Streaming, Survey and Setup data acquired prior to activating the DBS system, standard machine learning algorithms and deep learning methods will be used to predict which contact point will be used for stimulation at 6 months (the reference standard) and to model the effect of DBS at 6 months (defined as dichotomous improvement of bradykinesia, tremor and dyskinesia subscores of the MDS-UPDRS part III). For the standard machine learning models analysed at the group level, the spectral power and the volatility of each frequency bin will be extracted from Streaming, Timeline and Event data and used as features for contact point classification analysis. The Events data will be categorised according to its corresponding clinical, medication and stimulation state, aided by their concurrent kinematic tremor and dyskinesia scores obtained from the smartwatch measurements. The spectral power and the volatility of each frequency bin will be used to model the presence of the respective symptoms across participants, using interpretable machine learning algorithms, such as support vector machines and random forest models. If necessary, more advanced machine learning models, such as Extreme Gradient Boosting (XGBoost),^59^ will be explored to enhance prediction accuracy. XGBoost performs particularly well in handling noisy data, which is common in real-world data such as those used in the current study.^59^ For the deep learning models, specifically convolutional neural networks (CNNs), the classification of symptom states and the prediction of contact point used for stimulation are performed by using minimally processed Streaming data as input. CNNs enable the automatic determination of feature sets. InceptionTime,^60^ for example, is a CNN model specifically developed for time series classification, making it particularly suitable for identifying temporal patterns in the neuronal and kinematic data obtained in this study.^60^ These deep learning methods will also be used to develop patient-specific models to generate ‘neuronal fingerprints’ for individual symptom profiles. Patient-specific analyses do not require data normalisation. The predictive validity, in terms of sensitivity, specificity and the area under the receiver operating characteristic curve, of resulting individual neuronal fingerprints will be assessed through k-fold cross validation.

### Data management

Data concerning neurological evaluation and neurological rating scales will be kept in the case record forms in a trial master file and coded by case record number. The changes in medication and stimulation parameters will be recorded in electronic case record forms. A scalable, compliant with the US Health Insurance Portability and Accountability Act, cloud-based data platform, developed by the company Rune Labs, will be used to time-synchronise and aggregate the multimodal dataset acquired in the AI-DBS study.^61^

The kinematic smartwatch data is deidentified and stored under the specific participant code at the Rune Labs platform. This platform also allows monitoring of participant adherence to kinematic data collection. After 1 week of missing data, participants are contacted by phone to inquire about possible causes of missing data and to solve any underlying issues. This phone call also serves as a reminder for participants to adhere to their diaries.

All neuronal data, including patient diaries, will be temporarily stored in the Percept PC DBS system before it is deidentified and manually uploaded onto the platform for synchronisation after each visit. Timeline data are overwritten after precisely 2 months.^62 63^ During the first 6 months after DBS surgery, patients usually have regular clinical visits every 6–8 weeks. Neuronal data temporarily stored in the Percept neurostimulator is automatically retrieved during these clinical visits and made available for download and archiving by the study staff. If the time interval between standard care visits is longer than 2 months, additional study visits may be scheduled to ensure the retrieval and preservation of Timeline data. The Rune Labs platform is only used to store data. All analyses will be performed within the protected internal IT infrastructure of the Amsterdam UMC. For data processing, kinematic and neuronal data points that deviate more than 5 SD from the mean using a moving time window of 1 week will be omitted to filter out noisy data and ensure the accuracy of subsequent analyses.

### Study challenges

The protocol comes with a number of challenges. Although obtaining smartwatch and neuronal data from patients in their home environment might provide valuable insights, lack of contextual understanding potentially hinders interpretation. This poses a challenge for both the correlation analyses and for the application of supervised machine learning and deep learning techniques. Despite the promoting advances of the AI-based analysis, these approaches require labelled data to discern patterns and make accurate predictions, necessitating a comprehensive understanding of the context in which the data are collected. A potential source of noise in the collected data arises from the patient-reported symptom diaries. As these rely on subjective self-assessment, they are susceptible to inaccuracies due to participants’ varying perceptions, potential misunderstanding or incomplete recollection of symptoms. This subjectivity may affect the consistency and reliability of the data. However, reliability will be enhanced by providing clear instructions to participants and their caregivers, including concrete examples of when to log specific events. Additionally, event categories will be individually tailored in collaboration with each participant, using event labels designed to be intuitive for the participant, which will improve accuracy in self-reporting. During the study visits, the relevance of the event types will be evaluated, and any emerging symptoms will be identified, thereby ensuring ongoing alignment with the patient’s condition. Furthermore, the current study asks vulnerable and often elderly people with PD to wear a smartwatch, use its accompanying smartphone and make digital diary notes on the patient programmer, which raises some concerns regarding the practicality and acceptance of such technology among this study population. However, the patient programmer is specifically designed for use by this population, and fonts will be maximised on the smart devices to enhance readability. To reduce potential barriers to compliance and ensure more accurate reporting, participants (and their caregivers) will receive clear instructions, including detailed paper handouts illustrating step-by-step device handling. Automated reminders on participants’ smartwatches, regular monitoring of adherence, and ongoing support will also be provided. Furthermore, these instructional materials, along with additional screenshots, can be sent via email for remote assistance when necessary. We believe these measures will help mitigate the challenges associated with home-recorded measurements.

## Ethics and dissemination

The protocol and the informed consent form conform to the Geneva and Helsinki declarations and are approved by the medical research ethics committee of the Amsterdam UMC (registration number 2022.0368). All data concerning participants will be handled confidentially and in accordance with the General Data Protection Regulation and Data Protection Act 2018, which require data to be deidentified as soon as possible. A unique code will be assigned to each participant for identification throughout the study and data analysis. Monitoring will be performed by the Clinical Monitoring Center of the Clinical Research Unit of the AMC. The principal investigator will submit a summary of the progress of the trial to the accredited ethical board once a year. Results will be published in scientific journals and will be presented at scientific meetings.
