# Supplementary material for: AI-DBS study: protocol for a longitudinal prospective observational cohort study of patients with Parkinson’s disease for the development of neuronal fingerprints using artificial intelligence
Source: BMJ Open. 2025 May 16;15(5):e091563. doi: 10.1136/bmjopen-2024-091563 (PMC12086899; doi:10.1136/bmjopen-2024-091563)
Supplement: Supplementary file 1 [file bmjopen-2024-091563.reviewer_comments.pdf]

PEER REVIEW HISTORY

BMJ Open publishes all reviews undertaken for accepted manuscripts. Reviewers are asked to complete a checklist review form and are provided with free text boxes to elaborate on their assessment. These free text comments are reproduced below.

ARTICLE DETAILS

Title (Provisional)

The AI-DBS study: Protocol for a longitudinal prospective observational cohort study of Parkinson's disease patients for the development of neuronal fingerprints using artificial intelligence.

Authors

Stam, Mariëlle J.; de Neeling, Martijn G. J.; Keulen, Bart J.; Hubers, Deborah; de Bie, Rob M. A.; Schuurman, P. Rick; Buijink, Arthur W.G.; van Wijk, Bernadette C.M.; Beudel, Martijn

---

VERSION 1 - REVIEW

**Reviewer**	**1**
**Name**	**Isonaka, Risa**
**Affiliation**	**NIH**
**Date**	**26-Oct-2024**
**COI**	**None**

This protocol offers a promising approach to enhancing deep brain stimulation (DBS) for Parkinson's disease through AI-driven neuronal fingerprinting. However, to meet the journal's standards, several key areas require substantial revision:

1. English Language and Formality: The language should be refined to remove conversational phrasing, aiming for a formal, clear presentation that aligns with the journal's impact level.

2. Abbreviations: Ensure all abbreviations are defined upon first use, and only use standard, widely recognized abbreviations.

3. References: Update references, particularly on Parkinson's disease treatments and DBS, to reflect the latest research. Some key references appear outdated.

4. DBS Focus Justification: The rationale for focusing specifically on DBS and its effects on various Parkinson's symptoms (e.g., tremor, rigidity) should be expanded. This would clarify the study's unique contributions.

5. Novelty and Objectives: Explicitly highlight the innovative aspects of the study, particularly the use of AI to develop "neuronal fingerprints" for adaptive DBS, to clarify what sets this protocol apart from previous work.

6. Comparison Group: Consider including a comparison with a group receiving only levodopa treatment. This would provide insights into the added benefits or limitations of DBS over standard medication alone.

7. Genetic Considerations: Specify if genetic factors or subtypes of Parkinson's disease are considered in participant selection, as these may influence treatment response.

8. Follow-Up Process: Define the follow-up frequency and methods used for patient tracking. This would clarify how patient adherence and progress are monitored.

9. Incident Reporting: Include more details on how adverse events or incidents will be reported and managed to improve transparency.

10. Smartphone Requirement: If smartphone use is mandatory, clearly state this in the protocol, and consider discussing accessibility for older participants, as this may affect feasibility.

11. Expanded Discussion: Expand the discussion section to address these considerations, including the feasibility of frequent hospital visits, the patient burden, and the impact of continuous DBS on quality of life.

Reviewer 2

Name Giakoumettis, Dimitrios

Affiliation Agios Savvas General Cancer and Oncology Hospital of Athens, Neurosurgery

Date 20-Dec-2024

COI None

This is a succinct and highly scientific study protocol. The authors have explained the protocol sufficiently. However, I would like to see in the methodology section some extra info. I would respectfully recommend adding in the methodology, i) how will the team handle missing or noisy data in home-recorded measurements, ii) what was the rationale for choosing XGBoost and InceptionTime methods? Would this have to do that these methods can work well without standardization/normalization? Or is there another reason? iii) Would you consider normalization/standardization of your data?

VERSION 1 - AUTHOR RESPONSE

Reviewer: 1
Dr. Risa Isonaka, NIH
Comments to the Author:
This protocol offers a promising approach to enhancing deep brain stimulation (DBS) for Parkinson's disease through AI-driven neuronal fingerprinting. However, to meet the journal's standards, several key areas require substantial revision:

1. English Language and Formality: The language should be refined to remove conversational phrasing, aiming for a formal, clear presentation that aligns with the journal's impact level.

Thank you for this comment. We have refined the English language throughout the revised manuscript and have replaced all active voice phrases with passive voice for formality.

2. Abbreviations: Ensure all abbreviations are defined upon first use, and only use standard, widely recognized abbreviations.

We agree readability can be improved by avoiding as many abbreviations as possible. We have therefore removed several (scarcely used) abbreviations, and made sure all abbreviations are defined upon first use throughout the manuscript. For clarity, we have also added an abbreviations list in alphabetical order at the end of the manuscript.

3. References: Update references, particularly on Parkinson's disease treatments and DBS, to reflect the latest research. Some key references appear outdated.

Thank you for pointing this out. While we have included recent publications such as Deuschl et al. (2022) for guidelines on the treatment of Parkinson's disease, we acknowledge some of references are a little dated. However, we believe key works – like Little et al. (2013) pioneering aDBS, and Hauser et al. (2006) for treatment strategies, as well as Goetz et al. (2008), Jenkinson et al. (1997), Starkstein et al. (1992), and Beck et al. (1961) for clinical assessment scales – remain essential and widely cited due to their continued relevance and impact on current research and practice.

To strengthen the manuscript and reflect more recent advancements, we have added the meta-analysis by Nijhuis et al. (2021) to expand on Parkinson's disease treatments. For DBS as treatment, we now included the study of Vitek et al. (2020). We have also included Stanslaski et al. (2024) to the Introduction to incorporate the most recent developments in the area of DBS research.

References:

Beck AT, Ward CH, Mendelson M, Mock J, Erbaugh J. An inventory for measuring depression. Archives of general psychiatry. 1961;4(6):561-71.

Deuschl G, Antonini A, Costa J, Śmiłowska K, Berg D, Corvol JC, et al. European academy of neurology/Movement Disorder Society-European section guideline on the treatment of Parkinson's disease: I. Invasive therapies. European journal of neurology. 2022;29(9):2580-95.

Goetz CG, Tilley BC, Shaftman SR, Stebbins GT, Fahn S, Martinez-Martin P, et al. Movement Disorder Society-sponsored revision of the Unified Parkinson's Disease Rating Scale (MDS-UPDRS): scale presentation and clinimetric testing results. Movement disorders: official journal of the Movement Disorder Society. 2008;23(15):2129-70.

Hauser RA, McDermott MP, Messing S, Group PS. Factors associated with the development of motor fluctuations and dyskinesias in Parkinson disease. Archives of neurology. 2006;63(12):1756-60.

Jenkinson C, Fitzpatrick R, Peto V, Greenhall R, Hyman N. The Parkinson's Disease Questionnaire (PDQ-39): development and validation of a Parkinson's disease summary index score. Age and ageing. 1997;26(5):353-7.

Little S, Pogosyan A, Neal S, Zavala B, Zrinzo L, Hariz M, et al. Adaptive deep brain stimulation in advanced Parkinson disease. Annals of Neurology. 2013;74(March):449-57.

Nijhuis, F. A., Esselink, R., de Bie, R. M., Groenewoud, H., Bloem, B. R., Post, B., & Meinders, M. J. Translating evidence to advanced Parkinson's disease patients: a systematic review and meta-analysis. Movement Disorders. 2021;36(6), 1293-1307.

Stanslaski, S., Summers, R. L., Tonder, L., Tan, Y., Case, M., Raike, R. S., ... & ADAPT-PD Investigators. Sensing data and methodology from the Adaptive DBS Algorithm for Personalized Therapy in Parkinson's Disease (ADAPT-PD) clinical trial. npj Parkinson's Disease. 2024;10(1), 174.

Starkstein SE, Mayberg HS, Preziosi T, Andrezejewski P, Leiguarda R, Robinson R. Reliability, validity, and clinical correlates of apathy in Parkinson's disease. J Neuropsychiatry Clin Neurosci. 1992;4(2):134-9.

Vitek, J. L., Jain, R., Chen, L., Tröster, A. I., Schrock, L. E., House, P. A., ... & Starr, P. A. Subthalamic nucleus deep brain stimulation with a multiple independent constant current-controlled device in Parkinson's disease (INTREPID): a multicentre, double-blind, randomised, sham-controlled study. The Lancet Neurology. 2020;19(6), 491-501.

4. DBS Focus Justification: The rationale for focusing specifically on DBS and its effects on various Parkinson's symptoms (e.g., tremor, rigidity) should be expanded. This would clarify the study's unique contributions.

In the Introduction, we state that for patients who respond unsatisfactorily to regular pharmacological treatment, DBS is an effective advanced therapeutic option to manage medication-induced response fluctuations (lines 72–76). DBS not only reduces the duration and severity of symptoms during the OFF-drug phase but also decreases the severity and frequency of dyskinesias, ultimately improving the quality of life in PD patients (lines 80–84). Despite these benefits, not all PD patients with severe response fluctuations achieve optimal outcomes with DBS due to suboptimal motor improvements or stimulation-induced side effects (lines 85–87). This highlights the need to refine and enhance DBS therapy.

Importantly, the recent introduction of sensing-enabled neurostimulations such as the Percept allows for chronic recording of local field potentials (LFPs) in real-world conditions. This is a critical component of adaptive DBS development, which we are exploiting in the current protocol. Such chronic LFP recordings can only be conducted in patients who have DBS implanted, underscoring the essential role of this patient group in advancing adaptive DBS technologies.

5. Novelty and Objectives: Explicitly highlight the innovative aspects of the study, particularly the use of AI to develop "neuronal fingerprints" for adaptive DBS, to clarify what sets this protocol apart from previous work.

We agree we can improve on describing the novelty and innovative aspects of the study. The AI-DBS study will include the largest cohort of PD patients with sensing-enabled DBS to date. Recordings will be obtained in-clinic as well as during the patient's daily living for a prolonged period of time. The study will conduct a multimodal dataset, containing clinical data, kinematic data, neurophysiological data, for which advanced signal processing analyses (both at the group-level as well as at patient-level using machine- and deep learning methods) will be deployed to develop state-of-the-art adaptive DBS.

We have incorporated these novel- and unique aspects of the study into the Introduction by adding the following to the revised manuscript:

"To facilitate the translation of the technical advancements to clinical implementation of aDBS, the AI-DBS study will collect a high-resolution multi-modal dataset of, to our knowledge, the largest cohort of PD patients with sensing-enabled DBS to date, including recordings obtained in-clinic as well as during the patient's daily living for over 180 days. The latter is an important aspect in order to overcome the challenge of developing an aDBS control system able to react to patients' everyday actions, behaviors, and other relevant states, such as ON-OFF medication and ON-OFF stimulation (40, 41). A cohort of patients this large has the potential to unveil novel physiomarkers such as activity in other frequency bands alongside the well-established beta band or features in the time domain (41). We anticipate machine learning and deep learning techniques to be particularly useful for automatic discrimination between physiological and pathological neural activity (40). The methods also hold promise for advancing personalized treatment strategies, acknowledging the variability in disease symptoms across patients and the possibility of certain physiomarkers to not be applicable in all patients. The use of machine learning and deep learning in the AI-DBS study allows for individualizing the selection of (or the combination of) most effective or informative features concerning symptomatology or clinical status, hence following the concept of 'personalized

medicine'. The multifaceted data analysis approach employed in the AI-DBS study will enable the conduct of a future clinical trial comparing the neuronal fingerprint-based programming/DBS approach with conventional DBS, to further improve the clinical effect and efficiency of DBS for PD."

References:

40. Oliveira AM, Coelho L, Carvalho E, Ferreira-Pinto MJ, Vaz R, Aguiar P. Machine learning for adaptive deep brain stimulation in Parkinson's disease: closing the loop. Journal of Neurology. 2023;270(11):5313-26.

41. Neumann WJ, Gilron R, Little S, Tinkhauser G. Adaptive deep brain stimulation: from experimental evidence toward practical implementation. Movement disorders. 2023;38(6):937-48.

6. Comparison Group: Consider including a comparison with a group receiving only levodopa treatment. This would provide insights into the added benefits or limitations of DBS over standard medication alone.

Thank you for your suggestion. The aim of our study is to obtain a neurophysiological signature of patients' specific symptoms. The neurophysiological signature is obtained from brain recordings measured by the implanted DBS system. This means that for patients only treated with levodopa, we will not be able to obtain these brain recordings, therefore not allowing to create the neurophysiological signature. The experimental design and study aim do not ask for a control group.

Nevertheless, Parkinson's symptom severity (in two conditions: OFFmed and ONmed), quality of life, apathy, and depressive symptoms will be assessed prior to DBS surgery, i.e. when patients only receive levodopa treatment. In the Methods section "Clinical data", we write that these clinical data will be used to evaluate the effect of DBS at the end of the study period (during visit 5). Furthermore, kinematic data collected from the smartwatch will also be available during the one month before DBS surgery, when patients only receive levodopa treatment. This data is also available for comparison with the post-DBS situation. Still, the ultimate aim of the AI-DBS study is to improve DBS therapy by tailoring DBS into personalized medicine using patient-specific neuronal fingerprints, not to compare DBS therapy to levodopa treatment.

7. Genetic Considerations: Specify if genetic factors or subtypes of Parkinson's disease are considered in participant selection, as these may influence treatment response

In the previous version of the manuscript, the study population is defined as: "One hundred patients with PD who will be implanted with the Medtronic Percept™ PC neurostimulator for STN DBS in the care as usual setting will be included". Eligibility for DBS treatment is still determined based on the occurrence of motor fluctuations and levodopa responsiveness, regardless of patients' Parkinson's disease genetic subtype. This is because, so far, the literature on this does not provide evidence for different outcomes based on genetic subtype (Rizzone et al., 2018).

We specify these considerations in the revised version of the manuscript by:

"Eligibility for DBS treatment is determined based on the occurrence of motor fluctuations and levodopa responsiveness, regardless of patients' Parkinson's disease subtype. Only for diagnosing purposes, patients' genetic factors are considered, but not for the decision on being selected for DBS (42)."

References:

42. Rizzone, M. G., Martone, T., Balestrino, R., and Lopiano, L. (2018). Genetic background and outcome of Deep Brain Stimulation in Parkinson's disease. Parkinsonism Relat D 64, 8–19. doi: 10.1016/j.parkreldis.2018.08.006

8. Follow-Up Process: Define the follow-up frequency and methods used for patient tracking. This would clarify how patient adherence and progress are monitored.

Thank you for raising this question. Unfortunately, patient adherence to fill in a diary five times a day is not monitored real-time, but only reviewed during the in-clinic visits. Contrary, kinematic data collected through the Apple watch is monitored on the Rune Labs platform. Every week, the investigators monitor incoming data on the platform. Since StrivePD data can be saved locally on the Apple watch for a week, patients are only contacted by phone after one week of missing data. This phone call allows us to troubleshoot the kinematic data collection, but also enables us to monitor patients' adherence to the diary and/or remind patients to fill in the diary. We have added this information to paragraph: "Data management":

"This platform also allows monitoring of participant adherence to kinematic data collection. After one week of missing data, participants are contacted by phone to inquire about possible causes of missing data and to solve any underlying issues. This phone call also serves as a reminder for participants to adhere to their diaries."

9. Incident Reporting: Include more details on how adverse events or incidents will be reported and managed to improve transparency.

Thank you for raising this concern. To address this, we have included the following information in a revised Methods section "Patient burden and safety":

"Adverse events (AEs) reported spontaneously by participants or observed by the study staff will be systematically recorded. Serious adverse events (SAEs) associated with study-related procedures will be reported by the investigator without undue delay after obtaining knowledge of the events. SAEs resulting in death or considered life-threatening will be reported via the "ToetsingOnline" web portal to the accredited medical research ethics committee that approved the protocol. The initial report must be submitted within seven days of awareness, followed by an additional period of up to eight days for a detailed preliminary report. All other SAEs will be reported within 15 days of first knowledge by the staff. AEs will be monitored until they resolve or stabilize. Follow-up actions may involve additional tests, medical procedures, or referral to a general practitioner or specialist, according to the nature of the event. Reporting of SAEs is required until the end of the study within the Netherlands."

10. Smartphone Requirement: If smartphone use is mandatory, clearly state this in the protocol, and consider discussing accessibility for older participants, as this may affect feasibility.

Thank you for raising this concern. Whereas patients newly implanted with DBS are relatively young as compared to the general population of patients with Parkinson's disease, our study asks vulnerable and often elderly people to wear a smartwatch and use a smartphone. Although the required usage is limited, this still may impact feasibility of the study. In the manuscript, we indicate that patients have the right to withdraw consent at any time without citing reasons and without any consequences. We also indicate that patients have the option to withdraw consent from certain elements of the study. In a new paragraph of the Methods section in the revised version of the manuscript: "Study challenges", we further highlight this concern of asking vulnerable and often elderly people with Parkinson's disease to use smart devices, by:

"In addition, the current study asks vulnerable and often elderly people with PD to wear a smartwatch, use its accompanying smartphone, and make digital diary notes on the patient programmer, which raises some concerns regarding the practicality and acceptance of such technology among this study population."

11. Expanded Discussion: Expand the discussion section to address these considerations, including the feasibility of frequent hospital visits, the patient burden, and the impact of continuous DBS on quality of life.

Thank you for the suggestion to address the aspects of the study. The editor stated that a Discussion section is not part of the journal's formatting requirements for protocol articles. Therefore, we have either removed or moved the content to other sections within the revised version of the manuscript.

We have moved the information on the patient burden to the revised Methods section "Patient burden and safety":

"The administration of the different clinical scales in this study is part of the standard procedures in the Amsterdam UMC, and the study visits mostly coincide with regular clinical visits. Participation adds only slight discomfort of wearing a smartwatch, making small diary notes and undergoing a maximum of two extra testing visits. Therefore, the envisioned disadvantage of participation is limited. However, if patients experience excessive burden from participation, they also have the option to withdraw consent from certain elements of the study. The proposed protocol bears virtually no risks since readout of the neural recordings is safe and non-invasive. Although patients will not directly benefit from their participation, it is conceivable that in the medium to long term, the AI-DBS study might lead to improvements in (a)DBS treatment that will be to their benefit."

It is known that the longitudinal passive LFP recordings, i.e. BrainSense™ Timeline data, are overwritten after precisely two months (Sanger et al., (2024); Swinnen et al., (accepted)). During the first six months after DBS surgery, patients usually have regular clinical visits every 6-8 weeks. Neuronal data temporarily stored on the Percept neurostimulator is automatically retrieved during these clinical visits, and are available for download and archive to the study staff. If the time interval between standard care visits appears insufficient (i.e. longer than 2 months), additional study visits may be scheduled to secure retrieval and preservation of Timeline data. We incorporated this information on the avoidance of missing data also to the Methods section "Data management":

"Timeline data is overwritten after precisely two months (62, 63). During the first six months after DBS surgery, patients usually have regular clinical visits every 6-8 weeks. Neuronal data temporarily stored on the Percept neurostimulator is automatically retrieved during these clinical visits and made available for download and archiving by the study staff. If the time interval between standard care visits is longer than two months, additional study visits may be scheduled to ensure the retrieval and preservation of Timeline data."

References:

62. Sanger ZT, Henry TR, Park MC, Darrow D, McGovern, RA, & Netoff TI. Neural signal data collection and analysis of Percept™ PC BrainSense recordings for thalamic stimulation in epilepsy. Journal of neural engineering. 2024;21(1):012001.

63. Swinnen B, Buijink AWG, Stam MJ, Hubers D, Neeling MGJ, Keulen B, Morgante F, Van Wijk BCM, De Bie RMA, Ricciardi L, Litte S, Beudel M. Technical pitfalls and tips for using Percept™ BrainSense™ in DBS clinical practice and research. Journal of neural engineering. (Accepted).

Reviewer: 2
Dr. Dimitrios Giakoumettis, Agios Savvas General Cancer and Oncology Hospital of Athens
Comments to the Author:
This is a succinct and highly scientific study protocol. The authors have explained the protocol sufficiently. However, I would like to see in the methodology section some extra info. I would respectfully recommend adding in the methodology,

i) how will the team handle missing or noisy data in home-recorded measurements,

Thank you for raising this question. In the previous version of the manuscript, we acknowledged the challenge of obtaining data from patients in their home environment. While such data is expected to provide valuable insights, it also provides data devoid of contextual understanding, potentially hindering interpretation.

Patient adherence to fill in a diary five times a day is not monitored real-time, but only reviewed during the in-clinic visits. Contrary, kinematic data collected through the Apple watch is monitored on the Rune Labs platform. Every week, the investigators monitor incoming data on the platform. Since StrivePD data can be saved locally on the Apple watch for a week, patients are only contacted by phone after one week of missing data. This phone call allows us to troubleshoot the kinematic data collection, but also enables us to monitor patients' adherence to the diary and/or remind patients to fill in the diary. We have incorporated this information to paragraph: "Data management":

"This platform also allows monitoring of participant adherence to kinematic data collection. After one week of missing data, participants are contacted by phone to inquire about possible causes of missing data and to solve any underlying issues. This phone call also serves as a reminder for participants to adhere to their diaries."

It is known that the longitudinal passive LFP recordings, i.e. BrainSense^{TM} Timeline data, are overwritten after precisely two months (Sanger et al., (2024); Swinnen et al., (accepted)). During the first six months after DBS surgery, patients usually have regular clinical visits every 6-8 weeks. Neuronal data temporarily stored on the Percept neurostimulator is automatically retrieved during these clinical visits, and are available for download and archive to the study staff. If the time interval between standard care visits appears insufficient (i.e. longer than 2 months), additional study visits may be scheduled to secure retrieval and preservation of Timeline data. We incorporated this information on the avoidance of missing data also to the Methods section "Data management":

"Timeline data is overwritten after precisely two months (62, 63). During the first six months after DBS surgery, patients usually have regular clinical visits every 6-8 weeks. Neuronal data temporarily stored on the Percept neurostimulator is automatically retrieved during these clinical visits and made available for download and archiving by the study staff. If the time interval between standard care visits is longer than two months, additional study visits may be scheduled to ensure the retrieval and preservation of Timeline data."

For handling noisy data, kinematic and neuronal data points deviating more than five standard deviations from the mean will be omitted. We also incorporate this into the revised Methods section "Data management", by:

"For data processing, kinematic and neuronal data points that deviate more than five standard deviations from the mean will be omitted to filter out noisy data and ensure the accuracy of subsequent analyses."

Finally, a potential source of noise in the collected data arises from the patient-reported symptom diaries. As these rely on subjective self-assessment, they are susceptible to inaccuracies due to patients' varying perceptions, potential misunderstanding, or incomplete recollection of symptoms. This subjectivity may affect the consistency and reliability of the data. We included this in the Methods section "Study challenges", by:

"A potential source of noise in the collected data arises from the patient-reported symptom diaries. As these rely on subjective self-assessment, they are susceptible to inaccuracies due to participants' varying perceptions, potential misunderstanding, or incomplete recollection of symptoms. This subjectivity may affect the consistency and reliability of the data."

The challenges associated with home-recorded measurements, i.e. patients' compliance and accurate reporting, are mitigated by providing clear instructions to patients and their care partners, regularly monitoring patients' adherence, and providing ongoing support. We also incorporate this information in the Methods section "Study challenges":

"*Clear instructions to participants (and their care partners), regularly monitoring participants' adherence, and ongoing support are provided to reduce potential barriers to participants' compliance and to ensure more accurate reporting. We believe these measures will help mitigate the challenges associated with home-recorded measurements.*"

References:

62. Sanger ZT, Henry TR, Park MC, Darrow D, McGovern, RA, & Netoff TI. Neural signal data collection and analysis of Percept™ PC BrainSense recordings for thalamic stimulation in epilepsy. Journal of neural engineering. 2024;21(1):012001

63. Swinnen B, Buijink AWG, Stam MJ, Hubers D, Neeling MGJ, Keulen B, Morgante F, Van Wijk BCM, De Bie RMA, Ricciardi L, Litte S, Beudel M. Technical pitfalls and tips for using Percept™ BrainSense™ in DBS clinical practice and research. Journal of neural engineering. (Accepted).

ii) what was the rationale for choosing XGBoost and InceptionTime methods? Would this have to do that these methods can work well without standardization/normalization? Or is there another reason?

Thank you for this comment. We acknowledge the lack of clear rationale for choosing these methods as examples in the previous version of the manuscript.

Our intended approach is to initially focus on interpretable machine learning algorithms, such as support vector machines and random forests, given their capacity to provide greater transparency and insights into the decision-making process. However, if enhanced prediction accuracy is required, we intend to incorporate more advanced algorithms such as Extreme Gradient Boosting (XGBoost) (Chen et al., 2016). XGBoost is suggested because of its strong performance in predictive modeling. While not specifically designed for feature extraction, XGBoost excels at managing noisy data, which is common in real-world datasets like ours. Its effectiveness in modeling complex relationships and its robustness to data variability made it a suitable choice. Additionally, it works well without the need for extensive standardization or normalization, which further simplifies its application.

With regard to deep learning, the rationale lies in leveraging convolutional neural networks (CNNs) due to their ability to perform automatic feature extraction. InceptionTime is suggested as example of a CNN model, highly specialized in time series classification. This method is particularly suitable for identifying temporal patterns in time-dependent data, such as the neural and kinematic signals obtained in the AI-DBS study. InceptionTime has been shown to perform well in handling time series data without requiring extensive preprocessing, such as normalization or standardization, which is advantageous for complex multimodal datasets (Ismail Fawaz et al., 2020).

We have added these justifications to the revised manuscript for clarity, by:

"The spectral power and the volatility of each frequency bin will be used to model the presence of the respective symptoms across participants, utilizing interpretable machine learning algorithms, such as support vector machines and random forest models. If necessary, more advanced machine learning models, such as Extreme Gradient Boosting (XGBoost) (59), will be explored to enhance prediction accuracy. XGBoost performs particularly well on handling noisy data, which is common in real-world data like obtained in the current study (59). For the deep learning models, specifically convolutional neural networks (CNNs), the classification of symptom states and the prediction of contact point used for stimulation are performed by using minimally processed Streaming data as input. CNNs enable the automatic determination of feature sets. InceptionTime (60), for example, is a CNN model specifically developed for time

series classification, making it particularly suitable for identifying temporal patterns in the neuronal and kinematic data obtained in this study (60).”

References

59. Chen T, Guestrin C. XGBoost: A Scalable Tree Boosting System. Proceedings of the 22nd ACM SIGKDD International Conference on Knowledge Discovery and Data Mining. 2016:785–794.

60. Ismail Fawaz IH, Lucas B, Forestier G, Pelletier C, Schmidt DF, Weber J, et al. InceptionTime: Finding AlexNet for time series classification. Data Mining and Knowledge Discovery. 2020;34(6):1936-62.

iii) Would you consider normalization/standardization of your data?

Thank you for pointing this out. Regarding data normalization, we consider different approaches for the two data analysis components.

For the first approach, which involves conventional data analyses to correlate different data modalities with (changes in) clinical outcomes on a group level, normalization will be applied where appropriate. Neuronal Timeline data and kinematic scores are Z-transformed to enable meaningful comparisons across patients. Relative full spectrum PSDs will be obtained by normalizing the power at each frequency of the absolute full spectrum PSD to the total summed power of that specific spectrum. Additionally, we aim to balance the contributions of individual patients by accounting for data availability (e.g., the number of recorded Events and adherence to wearing the smartwatch) to avoid bias in group-level comparisons. This consideration is specified by:

“Where appropriate, Z-transformation will be applied to both neuronal Timeline data and kinematic scores, to enable meaningful group-level correlations. Relative full spectrum PSDs will be obtained by normalizing the power at each frequency of the absolute full spectrum PSD to the total summed power of that specific spectrum. Additionally, the contributions of individual participants will be balanced by down sampling, taking into account data availability (e.g., the number of recorded Events and adherence of wearing the smartwatch) to avoid bias in group-level comparisons.”

For the second approach, which focuses on developing patient-specific neuronal fingerprints using machine learning and deep learning methods, normalization will generally not be applied beyond outlier removal. This decision aligns with the strengths of the selected methods (e.g., XGBoost and InceptionTime), which are robust to data variability and do not inherently require normalization or standardization. These methods process minimally preprocessed data to maintain the integrity of individual patient-specific patterns, which are essential for modeling unique symptom profiles. We incorporated this consideration by:

“Patient-specific analyses do not require data normalization.”

VERSION 2 - REVIEW

**Reviewer**	**1**
**Name**	**Isonaka, Risa**
**Affiliation**	**NIH**
**Date**	**06-Mar-2025**
**COI**	

The manuscript has significantly improved in clarity and structure. I have a few remaining comments that should be addressed before publication:

• The figure legends should provide a clearer description of what each figure is showing, ensuring that a reader can understand the main message without having to refer back to the text. Consider briefly summarizing key data points presented in each figure.

• The manuscript does a good job of defining the study's novelty, but reinforcing the concept of “neuronal fingerprints” earlier in the introduction would improve readability.

• The rationale for using XGBoost and InceptionTime is provided, but a bit more clarity on why these were chosen over other models would be helpful. Were alternative methods tested before selecting these models? If so, were any comparative results available to support the choice?

• The revised manuscript details the handling of missing smartwatch and neuronal data, which is helpful. However, for the extreme data points (beyond five standard deviations), is there a secondary check to confirm if these are true anomalies or collection errors before exclusion?

• The manuscript states that missing data is handled via weekly monitoring and phone call reminders. Are there any additional strategies, such as automated reminders, training sessions, or caregiver support, to ensure better adherence?

• The manuscript acknowledges the challenges elderly participants may face in using smart devices. Were any accommodations made, such as simplified interfaces, larger fonts, or caregiver assistance, to facilitate participation?

• The subjective nature of patient diaries is noted as a limitation. Are there any strategies (such as cross-validation with smartwatch data) to mitigate potential biases in self-reported symptoms?

VERSION 2 - AUTHOR RESPONSE

Reviewer: 1
Dr. Risa Isonaka, NIH
Comments to the Author:

The manuscript has significantly improved in clarity and structure. I have a few remaining comments that should be addressed before publication:

- The figure legends should provide a clearer description of what each figure is showing, ensuring that a reader can understand the main message without having to refer back to the text. Consider briefly summarizing key data points presented in each figure.

Thank you for your valuable feedback. We agree that the description of Figure 1 was too brief, and we have expanded the figure legend to include relevant details directly visible in the figure. This ensures that readers can better interpret the figure without necessarily referring to the main text. Regarding Figure 2, we believe the existing description sufficiently conveys the key points, i.e. an example of the diary events that can be entered by the participant. Please let us know if additional adjustments would be beneficial. We have revised the description of Figure 1 into:

*“**Figure 1.** Schematic representation of the different forms of data collection during the study. The horizontal axis represents the approximate 7-month time line during which a patient is enrolled in the study. The six study visits to the hospital are indicated at the top of the figure together with the moment of DBS implantation (black vertical line). Participants will wear a smartwatch with built-in inertial sensors throughout the entire study period from which estimates of tremor and dyskinesia severity are determined (black horizontal line in the blue row, ‘Kinematic data’). Participants’ motor symptom severity will be determined while the DBS is on and participants are on their regular medication during visits 2-4, and in four different conditions (DBS off/on and off/on medication) during visit 5 (black triangles in cyan row, ‘Clinical data’). Local field potential recordings (red row, ‘Neuronal data’) will be conducted from DBS implantation onwards, not only during visits 1–5 (black triangles) but also passively at home (black horizontal line) and at given moments throughout the day at home (black dots, moments are randomly indicated in the figure). Participants will indicate how they are feeling at these same specific moments of the day (black dots in the yellow row, ‘Diary data’). Adjustments in stimulation parameters and/or medication schedule as part of care as usual DBS procedure will be registered throughout the study (moments are randomly indicated with the black arrows in the grey row, ‘Stimulation + Medication changes’).”*

- The manuscript does a good job of defining the study's novelty, but reinforcing the concept of “neuronal fingerprints” earlier in the introduction would improve readability.

Thank you for pointing this out. In this revised version of the manuscript, we introduce the concept of the “neuronal fingerprints” earlier in the Introduction, by:

“This highlights the need for a personalized ~~stimulation~~ algorithm that adjusts stimulation based on a patient- and symptom-specific profile of pathological neuronal activity, i.e. a “neuronal fingerprint”.

- The rationale for using XGBoost and InceptionTime is provided, but a bit more clarity on why these were chosen over other models would be helpful. Were alternative methods tested before selecting these models? If so, were any comparative results available to support the choice?

Thank you for raising this question.
In response to the concern raised by the second reviewer during first revision, we acknowledged the lack of clear rationale for choosing these methods as examples in the initial version of the manuscript. Our intended approach is to focus on interpretable machine learning algorithms, such as support vector machines and random forests, given their capacity to provide greater transparency and insights into the decision-making process. However, if enhanced prediction accuracy is required, we intend to incorporate more advanced algorithms such as Extreme Gradient Boosting (XGBoost) (Chen et al., 2016). XGBoost is suggested because of its strong performance in predictive modeling. While not specifically designed for feature extraction, XGBoost excels at managing noisy data, which is common in real-world datasets like ours. Its effectiveness in modeling complex relationships and its robustness to data variability made it a suitable choice. Additionally, it works well without the need for extensive standardization or normalization, which further simplifies its application.

With regard to deep learning, the rationale lies in leveraging convolutional neural networks (CNNs) due to their ability to perform automatic feature extraction. InceptionTime is suggested as an example of a CNN model, highly specialized in time series classification. This method is particularly suitable for identifying temporal patterns in time-dependent data, such as the neural and kinematic signals obtained in the AI-DBS study. InceptionTime has been shown to perform well in handling time series data without requiring extensive preprocessing, such as normalization or standardization, which is advantageous for complex multimodal datasets (Ismail Fawaz et al., 2020).

We have added these justifications to the previously revised manuscript clarifying the rationale of using XGBoost and InceptionTime:

“The spectral power and the volatility of each frequency bin will be used to model the presence of the respective symptoms across participants, utilizing interpretable machine learning algorithms, such as support vector machines and random forest models. If necessary, more advanced machine learning models, such as Extreme Gradient Boosting (XGBoost) [59], will be explored to enhance prediction accuracy. XGBoost performs particularly well on handling noisy data, which is common in real-world data like obtained in the current study [59]. For the deep learning models, specifically convolutional neural networks (CNNs), the classification of symptom states and the prediction of contact point used for stimulation are performed by using minimally processed Streaming data as input. CNNs enable the automatic determination of feature sets. InceptionTime [60], for example, is a CNN model specifically developed for time series classification, making it particularly suitable for identifying temporal patterns in the neuronal and kinematic data obtained in this study [60].”

Since both XGBoost as well as InceptionTime are provided as examples to indicate the type of advanced machine- and deep learning models, respectively, rather than an in-depth analysis of which algorithm to use, no alternative methods were tested before suggesting these algorithms.

References

59. Chen T, Guestrin C. XGBoost: A Scalable Tree Boosting System. Proceedings of the 22nd ACM SIGKDD International Conference on Knowledge Discovery and Data Mining. 2016:785–794.

60. Ismail Fawaz IH, Lucas B, Forestier G, Pelletier C, Schmidt DF, Weber J, et al. InceptionTime: Finding AlexNet for time series classification. Data Mining and Knowledge Discovery. 2020;34(6):1936-62.

- The revised manuscript details the handling of missing smartwatch and neuronal data, which is helpful. However, for the extreme data points (beyond five standard deviations), is there a secondary check to confirm if these are true anomalies or collection errors before exclusion?

Thank you for raising this concern. Based on our experience with pilot data, values exceeding five standard deviations solely represent anomalies rather than true physiological variations. To refine data handling, we will apply this threshold using a moving time window of one week, ensuring that deviations are assessed relative to local rather than global distributions. This refinement has now been incorporated into the revised manuscript, as detailed below. While cross-checking with simultaneously recorded data modalities could provide additional context, interpreting data in an unsupervised home environment, i.e. without contextual understanding, remains challenging. However, if both neuronal and smartwatch data exhibit outliers at the same moment, this is more likely indicative of a shared artifact rather than a true physiological event. Given the impracticality of manually verifying or visually inspecting our large dataset and the minimal expected impact of rare false positives, we consider the threshold of five standard deviations sufficient to exclude improbable values while maintaining data integrity.

“For data processing, kinematic and neuronal data points that deviate more than five standard deviations from the mean using a moving time window of one week will be omitted to filter out noisy data and ensure the accuracy of subsequent analyses.”

- The manuscript states that missing data is handled via weekly monitoring and phone call reminders. Are there any additional strategies, such as automated reminders, training sessions, or caregiver support, to ensure better adherence?

Thank you for raising this concern. In the previous version of the Methods section “5.5. Study challenges”, we indicate that the challenge of obtaining data from patients in their home environment, i.e. patients’ compliance and accurate reporting, will be mitigated by providing clear instructions to patients and their caregivers, regularly monitoring patients’ adherence, and providing ongoing support.

Furthermore, in "5.3. Data collection", we briefly mention that patients will receive automated reminders on their smartwatch to remind them to indicate how they are feeling. We have emphasized this aspect in the revised version of Methods section "5.5. Study challenges", by:

"To reduce potential barriers to compliance and ensure more accurate reporting, participants (and their caregivers) will receive clear instructions, including detailed paper handouts illustrating step-by-step device handling. Automated reminders on participants' smartwatches, regular monitoring of adherence, and ongoing support will also be provided. Furthermore, these instructional materials, along with additional screenshots, can be sent via email for remote assistance when necessary. ~~Clear instructions to participants (and their care partners), regularly monitoring participants' adherence, and ongoing support are provided to reduce potential barriers to participants' compliance and to ensure more accurate reporting.~~ We believe these measures will help mitigate the challenges associated with home-recorded measurements."

- The manuscript acknowledges the challenges elderly participants may face in using smart devices. Were any accommodations made, such as simplified interfaces, larger fonts, or caregiver assistance, to facilitate participation?

Thank you for pointing this out. The patient programmer is designed to be used by this same patient population, having a simplified interface and large fonts. For the AI-DBS study, the default fonts on the smartphone and smartwatch will be set to the largest as possible. Additionally, clear instructions about the use of the smart devices will be provided to participants and caregivers, including paper handouts showing intermittent steps of device handling. These same documents and screenshots can be sent through mail when providing remote support. We have added this information to Methods section "5.5. Study challenges". Since the information slightly coincides with the information provided in our answer to your previous comment, the revised Methods section "5.5. Study challenges" below is partly duplicated from our previous answer:

"~~In addition~~Furthermore, the current study asks vulnerable and often elderly people with PD to wear a smartwatch, use its accompanying smartphone, and make digital diary notes on the patient programmer, which raises some concerns regarding the practicality and acceptance of such technology among this study population. However, the patient programmer is specifically designed for use by this population, and fonts will be maximized on the smart devices to enhance readability. To reduce potential barriers to compliance and ensure more accurate reporting, participants (and their caregivers) will receive clear instructions, including detailed paper handouts illustrating step-by-step device handling. Automated reminders on participants' smartwatches, regular monitoring of adherence, and ongoing support will also be provided. Furthermore, these instructional materials, along with additional screenshots, can be sent via email for remote assistance when necessary. ~~Clear instructions to participants (and their care partners), regularly monitoring participants' adherence, and ongoing support are provided to reduce potential barriers to participants' compliance and to ensure more accurate reporting.~~ We believe these measures will help mitigate the challenges associated with home-recorded measurements."

- The subjective nature of patient diaries is noted as a limitation. Are there any strategies (such as cross-validation with smartwatch data) to mitigate potential biases in self-reported symptoms?

Thank you for your comment. While acknowledging the limitation of self-reported symptom tracking, we will enhance reliability and mitigate potential biases by providing participants and their caregivers with clear instructions, including concrete examples of when to log specific events. Additionally, event categories will be individually tailored in collaboration with each participant, ensuring they align with their specific symptoms and experiences. Event labels will be designed to be intuitive for the patient, improving accuracy in self-reporting. Regular follow-ups will be conducted to assess whether event types remain relevant and to identify any emerging symptoms, ensuring ongoing alignment with the patient's condition. We have added this information to the revised Methods section "5.5. Study challenges", by:

“A potential source of noise in the collected data arises from the patient-reported symptom diaries. As these rely on subjective self-assessment, they are susceptible to inaccuracies due to participants’ varying perceptions, potential misunderstanding, or incomplete recollection of symptoms. This subjectivity may affect the consistency and reliability of the data. However, reliability will be enhanced by providing clear instructions to participants and their caregivers, including concrete examples of when to log specific events. Additionally, event categories will be individually tailored in collaboration with each participant, using event labels designed to be intuitive for the participant, which will improve accuracy in self-reporting. During the study visits, the relevance of the event types will be evaluated and any emerging symptoms will be identified, thereby ensuring ongoing alignment with the patient’s condition.”

VERSION 3 - REVIEW

**Reviewer**	**1**
**Name**	**Isonaka, Risa**
**Affiliation**	**NIH**
**Date**	**17-Apr-2025**
**COI**	

The authors have addressed all previous comments. I believe the manuscript is suitable for publication in this journal.
